# “*Asthma is a* very *bully disease*” – patient experiences of living with chronic respiratory diseases in Cape Town, South Africa

**DOI:** 10.1186/s12939-023-02002-5

**Published:** 2023-09-14

**Authors:** Marie Stolbrink, Chantel Streicher, Khanyisa Mcimeli, Brian Allwood, Kevin Mortimer, Martha Chinouya

**Affiliations:** 1 Clinical Sciences, Liverpool School of Tropical Medicine, Liverpool, UK; 2 Division of Pulmonology, Department of Medicine, Stellenbosch University, Cape Town, South Africa; 3 Department of Psychology, Faculty of Arts and Social Sciences, Stellenbosch University, Stellenbosch, South Africa; 4 Faculty of Arts and Social Sciences, Stellenbosch University, Stellenbosch, South Africa; 5 Division of Pulmonology, Department of Medicine, Stellenbosch University and Tygerberg Hospital, Cape Town, South Africa; 6 Cambridge Africa, University of Cambridge, Cambridge, UK; 7 School of Clinical Medicine, College of Health Sciences, University of KwaZulu-Natal, Durban, South Africa; 8 Liverpool University Hospitals NHS Foundation Trust, Liverpool, UK; 9 Faculty of Education, Liverpool School of Tropical Medicine, Liverpool, UK

**Keywords:** Experience, Semi-structured interview, Cape Town, South Africa, Qualitative research, Chronic respiratory disease, Asthma, COPD, Healthcare

## Abstract

**Background:**

Chronic respiratory diseases are common in Cape Town, South Africa. Yet the experiences of how adults with these conditions, such as asthma or COPD (chronic obstructive pulmonary disease), negotiate the health system are poorly understood. Qualitative methodology lends itself to investigate this question.

**Aim of study:**

To explore the “emic” experiences of adults with CRDs in Cape Town when they were negotiating the health system using semi-structured interviews.

**Methods:**

Interviews were conducted following informed consent with purposively sampled adults who had attended public hospitals in Cape Town with chronic respiratory disease flare-ups. This work was nested in the quantitative “Diagnosing Airways Disease” study. The topic guide explored patients’ experiences of accessing healthcare including receiving and interpretations of the diagnosis and management, and impacts on daily life. Interviews were conducted in Afrikaans, isiXhosa, or English; transcribed, and translated into English and thematically analysed until saturation.

**Results:**

Thirty-two interviews (16 in Afrikaans, 8 in isiXhosa, 8 in English) were completed in 2022. 17 women and 15 men participated. Most participants were older than 50 years (25/32), and most were unemployed (13/32) or retired (11/32).

The identified themes were: Perceived causes of illness; experiences of healthcare; perceived risks and barriers when accessing healthcare; and impact on earnings. The perceived causes of their illness and risks were structural, and included air pollution, poor quality housing, occupational exposures, limited healthcare services, and fear of violence. These factors led to self-treatment, sharing of medicines, and delay in receiving a diagnosis. Many paid privately for treatments or services to overcome identified shortcomings of the public healthcare system, and many reported additional significant indirect costs. Being ill had a profound impact on income. The identified themes were explored through the lens of “structural violence”, where “social structures stop individuals … from reaching their full potential” (Galtung, 1969).

**Conclusion:**

In Cape Town structural elements such as stretched healthcare professionals, insufficiently enforced policies on e.g., housing or work-place exposures, poverty and crime made it difficult for participants to successfully navigate their illness experience. It forced some to pay out of pocket to receive perceived better healthcare privately.

**Supplementary Information:**

The online version contains supplementary material available at 10.1186/s12939-023-02002-5.

## Background

Non-communicable diseases (NCDs) cause significant morbidity and mortality globally, and those living in poverty and in low- and middle-income countries (LMICs) are disproportionately affected [1]. Chronic respiratory diseases (CRDs), and especially diseases affecting the airways such as asthma or chronic obstructive pulmonary disease (COPD), are major NCDs and cause challenges for LMICs. Healthcare systems in LMICs are historically set up and funded for episodic, acute healthcare provision, however, NCDs require life-long treatments and regular interactions with the healthcare system [2, 3]. Patients with CRDs may therefore struggle to access appropriate and timely treatment or be hindered by costs [1, 3].

The symptoms of CRDs can be well controlled when medicines are taken regularly and correctly in a supportive environment, however, patients may experience episodic flare-ups which may be life-threatening and require emergency treatment [4, 5]. In South Africa basic, effective inhaled and tablet medicines for CRDs are provided for free or heavily subsidised. Despite this, South Africa has a high prevalence of asthma symptoms and the third highest asthma mortality among LMICs worldwide [4, 6].

CRDs are medicalised conditions that require treatment and an accurate diagnosis. Conrad (1992) states that medicalisation “consists of defining a problem in medical terms, using medical language to describe [it], adopting a medical framework to understand [it], or using a medical intervention to "treat" it” [7]. Those with CRDs may aim to gain the “sick role”. The “sick role” means to “achieve recognition of ones suffering [by others and society] and … a social license to be exempt from particular duties for a given period of time” [8]. Being recognised as being “sick” could, for example, lead to a diagnosis, access to medications, adjusted work duties or financial support.

### What is known: experiences of living with CRDs in sub-Saharan Africa

In sub-Saharan Africa CRDs are often under-diagnosed and under-treated, medicines are unavailable or unaffordable, and there are misconceptions about treatments and prognosis [9–11]. In interviews with South Africans with chronic diseases (other than CRDs), waiting times, work commitments and self-treatment were given as reasons to miss appointments and collect medicines [12]. Parents of children with asthma reported medication costs as a barrier to biomedical treatment [13]. Data from healthcare workers and policy makers in Kenya, Malawi, Sudan, Tanzania and Uganda revealed that these health systems were ill-equipped to manage CRDs [14]. A South African audit showed that there were limited processes, deficient structures and poor outcomes for patients with asthma [15]. Yet most of the published work does not involve adults who are directly affected despite recognition that the voices of adults with CRDs in LMICs are essential to improve treatments and services [16].

### Setting

In South Africa, the interactions with the health system must be understood in the historical context. The distribution and provision of healthcare is complicated, and influenced by modern history and the Apartheid system, which ended in 1994 [17, 18]. The health system is split into public and private. More than two thirds of the population primarily use the government-funded public system, which is free [18]. Most health needs are attended to in community or primary care clinics but if the condition is more complex, patients are referred to district or tertiary care hospitals. Public facilities, and especially primary care clinics, have limited resources, experience understaffing and provide limited tests [18]. Some people choose to pay to additionally use private providers for medicines or consultations.

This study was conducted in Cape Town, an urban area in Western Cape, South Africa. Almost one in five households live in informal dwellings and townships, underdeveloped residential areas with origins in Apartheid [19]. CRDs are caused and exacerbated by smoking, air pollution and respiratory infections, such as tuberculosis. In South Africa about half of all men and roughly one in five women were or are current tobacco smokers, and smoking is more prevalent among poorer communities, such as those living in townships [20]. There is a high incidence of tuberculosis, with over 28,500 new cases in Cape Town alone in 2022 [21]. Cape Town is windy and has hot, dry, and dusty summers with wildfires, predisposing residents to air pollution. Air pollution levels regularly exceeded safe levels proposed by the World Health Organization (WHO) over a ten-year period [22]. There is legislation to minimise air pollution and harmful exposures in the workplace [23, 24]. Strict regulations during the COVID-19 pandemic, which included compulsory face masks and restriction of movements, were stopped in April 2022 [25].

The city is divided racially, geographically, and economically due to historical reasons, which is also represented in the language spoken in the household [26]. Most people in lower- and middle-income neighbourhoods speak Afrikaans, and most living in lower-income neighbourhoods and townships speak isiXhosa [27, 28]. English is spoken across economic groups. Almost one in four inhabitants of Cape Town is unemployed, and at least one in three lives in poverty [29, 30]. There is a high level of petty and serious crime, the province has the highest murder rate in the country [31].

The patients’ experiences are key in understanding how they interact with the healthcare system in Cape Town. By exploring this, we may be able to identify barriers to effective healthcare and how to overcome them. Semi-structured interviews, a qualitative method, explore personal experiences, attitudes and beliefs of individuals and therefore can be used to investigate this [32, 33].

## Methodology and methods

This was a qualitative study, which used an interpretivist epistemology to explore how participants interpreted their experiences of living with CRDs in Cape Town and their encounters within the health systems [34]. The explored “emic”, or “insider", experiences were investigated using semi-structured interviews. The manuscript was prepared using Standards for Reporting Qualitative Research (Appendix) [35].

### Ethical approval

Ethical approval was gained by Liverpool School of Tropical Medicine (reference 20–094) and Stellenbosch University (reference N20/11/124).

### Participant recruitment and consent

**Fig. 1:**
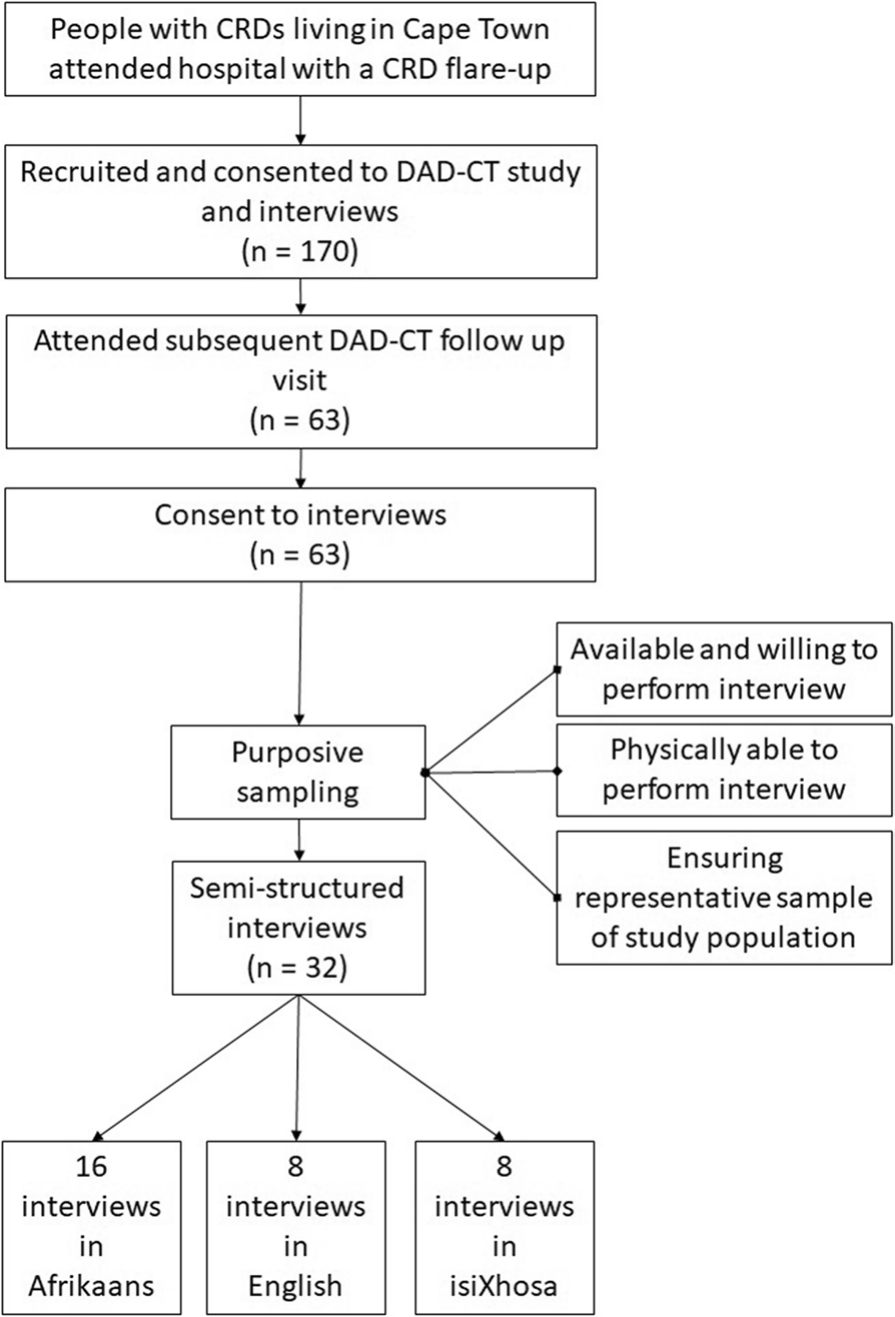
Overview of participant recruitment

This study was nested within the quantitative “Diagnosing Airways Disease in Cape Town (DAD-CT)” study, which was conducted in two public hospitals in Cape Town, located in a low-middle-income neighbourhood and a township. The cross-sectional DAD-CT study sought to establish a physiological diagnosis for patients with CRDs. The DAD-CT study recruited participants when they attended hospital with a flare-up, and then conducted clinical investigations at a single subsequent study visit at least 8 weeks later. Only participants who attended the subsequent study visit were invited to partake in semi-structured interviews (Fig. 1). Interview participants were sampled purposively to represent the DAD-CT study population (Table 1).

**Table 1:** Inclusion and exclusion criteria

Inclusion criteria	Exclusion criteria
- Participant in DAD-CT study - Attendance at subsequent study visit - Valid consent for participation in qualitative study - Representative sample of DAD-CT study population (gender, age, preferred language, employment status)	- Unavailable or unwilling to perform interview - Physically unable to perform interview

Written, informed, voluntary consent for the quantitative and qualitative study was gained upon original recruitment to the DAD-CT study. Participants read and received the participant information sheet for both aspects at recruitment. Participants re-confirmed their consent to the interviews at the subsequent visit, before interviews commenced. Interview participation had no impact on participation in the DAD-CT study or any other treatment. Participants were re-imbursed for their time and travel in line with Stellenbosch University guidelines.

### Approach to interviews

The semi-structured interviews followed a topic guide (Appendix) that was developed by MS and MC. It was informed by evidence emerging from literature on experiences of living with CRDs and experiences of the authors whilst working with those with CRDs [13, 36, 37]. It was structured around the experiences of receiving a diagnosis, understanding of diagnosis and treatment, impacts on daily life and improvement of services. The guide was reviewed iteratively between interviews and discussion within the research team. Revisions were conducted whilst interviews were being performed to improve it and the quality of the interviews. It was translated into Afrikaans and isiXhosa, back-translated into English to ensure correctness, and used by researchers who were fluent in the language.

Face to face semi-structured interviews were conducted in either Afrikaans, isiXhosa, or English, depending on the participants’ preferred language. CS (female, MA) conducted the interviews in Afrikaans, KMc (female, MPhil) in isiXhosa and MS (female, MA) in English. The interviewers had experience in conducting health interviews, had all been trained on using the topic guide and ethics. CS and KMc have social science backgrounds and no prior relationships with the participants. MS was a medical doctor and had met the participants during the DAD-CT study.

Interviews took place in a private room in a hospital, either when participants attended for the single visit, or when they attended specifically for the interviews. All interviews were recorded digitally, transcribed using the denaturalised approach and translated into English by the interviewer who conducted the interview. Reflexive notes were also kept by each interviewer during and after the interview to aid transcription and analysis.

### Data analysis

All analysis was performed on the English transcriptions. An interpretative approach with the framework method was used to analyse the transcripts [38–40]. The framework approach is a systematic way of analysing qualitative data within the research team and involves 1) transcription, 2) reading and familiarising with the interview, 3) coding, which is applying labels to the interpreted interview passage, 4) developing an analytical framework collaboratively with all the researchers, 5) then applying the agreed framework onto the transcripts [40]. Coding and interpretation of results was compared and discussed among the three researchers who conducted the interviews (CS, KMc, MS) and an external researcher (MC) to improve the consistency of coding, explore differences in interpretation and reduce the influence of personal reflexivity. This was iteratively repeated until no new themes emerged. Two researchers (CS and MS for Afrikaans and English interviews, KMc and MS for isiXhosa and English interviews) independently inductively analysed the transcribed interviews thematically using NVivo (version 12). Data collection proceeded until no new concepts emerged (thematic saturation). Finally, MS and MC used a funnelling approach to establish key themes [41]. These key themes were checked back against the original interview transcripts to ensure credibility and confirmability [42]. Below we discuss the emergent themes relating to causes of illness, experiences of healthcare, risks when accessing healthcare and impact of illness on earnings.

## Results

### Participant characteristics

A total of 32 interviews were conducted: 16 interviews in Afrikaans, 8 in isiXhosa and 8 in English (Table 2). Everyone who was approached took part in the qualitative study. 17 women and 15 men participated between March and December 2022. Most participants (25, 78%) were older than 50 years. Most were unemployed (13, 41%) or retired (11, 34%), only three participants (9%) were in employment. The interviews lasted between 25 and 62 min.

**Table 2:** Overview of interview participants

Attribute	Number (percentage)
**Gender**	
- Women	17 (53%)
- Men	15 (47%)
**Age**	
- > 50 years	25 (78%)
- ≤ 50 years	7 (22%)
**Preferred language**	
- Afrikaans	16 (50%)
- isiXhosa	8 (25%)
- English	8 (25%)
**Employment status**	
- Employed	3 (9%)
- Unemployed	13 (41%)
- Disability grant	5 (16%)
- Pensioner	11 (34%)
**Household composition**	
- Lives alone	3 (9%)
- Lives with others	29 (91%)
**Relationship status**	
- In relationship / married	14 (44%)
- Single / widowed	18 (56%)

### Identified themes

We identified the following four themes in our analysis: perceived causes of illness, experiences of healthcare, perceived risks and violence when accessing healthcare, and impact of illness on earnings. The included participant quotes are accompanied by their preferred language and gender.

#### Perceived causes of illness

The participants explained how or why they developed symptoms. Dusts, weather, fumes, housing, and work were identified as causes of illness but also triggers for flare-ups. This participant reported how moving to a colder, windier, and dustier area of Cape Town with their “shack” (rudimentary hut) impacted her and said:

*“[The shack] was cold and dusty because the place was new … It was windy, very windy that year we moved in. And the sand was coming up … So that is why I get worse.”* [Female speaker 6, English]

Fumes from burning scrap and animal waste were also reported as having direct effects on their health, as one participant described:

*“My neighbours they drive scrap … He burns the electrical wires, he burns, he has horses that are there that give off the smell. He has chickens. That's not good, so I'm just constantly with the asthma pump.”* [Female speaker 4, Afrikaans]

Participants recognised that poor quality housing impacted their health, yet they had no choice but to live there as it was free:

*“The house affects my health, but I am staying in it because it was freely given to us. The roof made of asbestos sweats, like it’s raining on the roof.” *[Male speaker 32, isiXhosa]

The relationship of work and the illness was complicated. Work was identified as a reason for their illness, for example by this participant:

*“The dust I work with also makes it worse and affects my lungs. So, my line of work also affected my lungs.”* [Male speaker 25, isiXhosa]

In theory there should be workplace regulations, yet some reported that there was a difference between what was supposed to and what really happened. This participant reported working despite being exposed to chemicals that worsened his illness:

*“When it came to the chemicals [my colleagues] would just warn me if they were going to be using this … oven cleaner … So, I would go and stand outside. Because the moment they spray it, and I get just the smell of it, my chest immediately starts to close ... Or what I would do is, if I am too busy, I would wear a mask. Just wear a mask over my face and then work.”* [Male speaker 21, English]

This could escalate to a conflict between working and being ill, as this participant explained:

*“[I was] manager in the butchery. I worked this way for about 23 years ... I got so sick I had to leave it because I didn’t have a choice; I was too weak to work. Because you don’t want to leave your job because financially you need money.”* [Male speaker 6, Afrikaans]

One participant elaborated that her choice was between being alive and having a job:

*“Why did I leave my job? I could just die [at work]. But at the end of the day, what choice did I have? One’s health comes first or one’s job comes first, then I choose my health.”* [Female speaker 15, Afrikaans]

Hence environmental and socio-economic factors were identified as contributors and causes of CRDs.

#### Experiences of healthcare and getting the diagnosis

Having a CRD had a profound impact on participants’ daily lives, as this lady explained:

“*Asthma is very bully. I’m telling you. It is a bully, bully, bully, bully sickness. It tells you what to do. Stop, do it, stop, don’t do this, don’t go, don’t walk fast, go slow, you can move fast, slow … The cost of the disease empties my pockets many times.”* [Female speaker 4, Afrikaans]

Once symptoms, such as breathlessness or wheeze, persisted many participants reported delay in receiving a diagnosis and treatment as they thought that their encounters with doctors was inadequate. Doctors were sometimes seen as not engaging or spending adequate time with them and a participant explained:

*“The way they examine, it’s not properly. They ask questions and you answer, and they give you pills. It doesn't work that way.”* [Female speaker 2, Afrikaans]

Diagnoses were changed over time, which also confused, and frustrated, patients. There were no examinations and participants reported being puzzled about their exact diagnosis. This participant had heard “COPD” before but did not understand what it meant:

*“According to the hospital's diagnosis, it's COPD, you know what it is? Chronic Pulmonary Blood Disease [sic]. But for me, it's more emphysema. Emphysema comes from smoking, so I think that's what it comes from, but the Pulmonary I don't know what it's coming from.”* [Male speaker 11, Afrikaans]

Due to the perceived limited time with healthcare professionals, participants also did not know how to take their medicines. One participant developed a flare-up because she did not realise that her inhaler was empty, as she had not been trained on how to use it:

*“For 2 days I was turning and clicking and all that. And then I realised it was empty.”* [Female speaker 19, English]

Participants would therefore bypass doctors completely and buy “pumps” (inhalers) in pharmacies, to self-treat, and thus possibly delay in seeking medical help as one participant explained:

*“No, never seen a doctor. Only the pharmacy and what they would give me over the counter.”* [Female speaker 19, English]

Many reported that their experience in larger hospitals was better than in local clinics. They reported that the staff was more attentive and better trained, and therefore provided better care, saying:

*“In [local clinic] they don't work right with people, they ignore you, they're doing their own thing. I don't know what their thought was, they did all the tests, but they couldn't tell me what was wrong with me. But here at [large hospital], I immediately knew what was going on with me.”* [Male speaker 11, Afrikaans]

This participant feared for his life in the local clinic due to limited knowledge about how to manage patients with acute asthma:

*“You wait there [local clinic] too long while you have an asthma attack … They do not even put you on oxygen while you are waiting. An asthma attack is painful. You can die there.”* [Male speaker 28, isiXhosa]

When participants attended the facilities with symptoms, such as cough or breathlessness, investigations primarily revolved around evaluating for tuberculosis. However, participants conveyed that lung function tests, which are needed to make a diagnosis of asthma or COPD, were not performed.

Participants reported that results of tests were not relayed to them which ultimately prevented them from understanding their own illness. However, when it was explained to them, there was relief in getting a name, or diagnosis, for their experiences. This participant explained:

*“When they tell me now, I have asthma, now I think with asthma you know your breathing isn't right you're having trouble walking, you're struggling to breathe. It's called asthma ... When I found out about the asthma it was more I understood why I was struggling to breathe.”* [Female speaker 4, Afrikaans]

Some therefore demanded appointments to understand what was happening with their health. Others arranged private healthcare to get answers, as they perceived the care to be better there:

*“I go to the [private] doctor and I leave the [public] clinic … I prefer to be with the [private] doctor who helps you correctly instead of going to the [public] clinic.”* [Female speaker 15, Afrikaans]

Participants were empowered by knowing about their health. It improved their wellbeing and self-management of their illness, as this participant portrayed:

*“I actually think my health is better if I find out more about it ... I like to know more about asthma … Because I always like to stay a step ahead and at least know what is going on, to have some idea of what the problem is and how I can cure or prevent it ... Because a lot of people they get an asthma attack and immediately it is a straight up asthma attack, and they can’t do nothing. Whereas I have learned to control it.”* [Male speaker 20, English]

Therefore, the experienced delays in receiving a diagnosis were related to limited time with healthcare professionals, inadequate investigations, and self-treatment. However, educating participants was an effective tool for self-management and empowerment.

#### Perceived risks and barriers when accessing healthcare

Those with CRDs regularly attend healthcare facilities for appointments or to collect medicines. They may also require urgent medical treatment when they experience a flare-up. However, access to the facilities was often difficult.

Many participants reported that they lived in”dangerous” neighbourhoods where walking was usually not possible due to safety concerns, as a female participant expressed:

*“If you are strong you can walk [to the hospital]. But is dangerous for us.”* [Female speaker 22, English]

Participants would consider being the victim of crime when accessing facilities and adjust their behaviour, as one participant explained:

*“You must not show them you are scared of them. He doesn’t know what you have. You mustn’t show them. You must just walk. I still walk to clinic. But sometimes I am scared. So, I take a taxi, or I talk to my friend to take me halfway.”* [Male speaker 24, English]

Even being on hospital or clinic grounds did not necessarily convey safety for this participant:

*“The junkies are [at the clinic] they search you.”* [Male speaker 28, isiXhosa]

Apart from the physical environment, clinics were also perceived as places where one may contract illnesses, or “become a victim” of disease, as this participant puts it:

*“I try to distance myself from places that have many people because I do not know who has it … so I could possibly be a victim easily.”* [Male speaker 27, isiXhosa]

After taking the risk and accepting the potential dangers of getting to the clinic, there was a risk that the clinic would be closed, as this participant experienced:

*“There is a day clinic around the corner. But when I went to the day clinic, that was around 3 o’clock, it was closed.”* [Male speaker 21, English]

Or that the clinic did not have the necessary treatment, as noted by this participant:

*“I went to the local health centre. It was a Sunday morning and after examining my X-rays there is nothing they can do for me and they sent me here [large hospital].”* [Female speaker 31, isiXhosa]

Another risk of collecting medicines and attending facilities was that due to long queues a single trip could take a whole day. This was burdensome and expensive. To avoid the problems of attending healthcare facilities, some patients reported sharing medicines with families, colleagues or friends as noted:

*“My aunt [who also has asthma], she gets from her medical aid, they send her every month a box of pumps and these medications. So sometimes she won’t use it because it is not necessary. She gives it to me. … She has 2 nebulisers. So she told me I can use the one and she has the other one. So it is just at my house when I need it. The liquid I would buy at the pharmacy, or if she has extra then she would obviously just give me some.”* [Male speaker 21, English]

This habit of medicine sharing was common amongst participants.

Participants would also pay for medicines out of pocket, in addition to, or instead of, a free prescription. This was often because they ran out of medicines before the next prescription was available, or because it was more convenient to go to a local pharmacy than to attend the health centre and queue.

One positive memory from the COVID-19 pandemic, was that medicines were delivered directly to people’s homes as this man explained:

*“Now they’re bringing you all the way home and it’s always ahead of time. That’s a good service.”* [Male speaker 10, Afrikaans]

People with CRDs may experience flare-ups of their condition, where they require urgent medical treatment ideally through an ambulance. Ambulances were free, so participants did not need to inconvenience others, as this participant discussed:

*“I don’t have transport myself. But there are people around that I can sometimes ask. But they are very busy. And usually [the flare-up] happens during the night. And people are sleeping. I don’t want to disturb people who are sleeping, they have to go to work tomorrow and then I call an ambulance.”* [Male speaker 30, isiXhosa]

Yet participants explained that there were often long delays after they called an ambulance, sometimes more than ten hours, as this participant narrated:

*“So [I waited for the ambulance] starting from 11, till 21:30 in the night. And you know, with asthma it is hard. You don’t think, you just want to breathe.”* [Female speaker 23, English]

One reason for the delays was that participants lived in unsafe areas, where gun and other violent crimes were common. Here, ambulances were targets for crime and therefore needed police escorts, which caused additional delays. This participant explained:

*“Because our area is in the red … there are ambulances now, but first they have to wait for a police car. Because our area is in the red because they shoot a lot there with us. It's also a long time we have to wait there, about 3, 4 hours before they get to us.”* [Female speaker 7, Afrikaans]

Generally private transport by car, from family, friends, or neighbours, was therefore the preferred choice. Participants would often pay or reimburse the drivers, like this participant:

*"When I go to the clinic I would say I feel safe in sense that there are people I can call to accompany me to the clinic. I ask my neighbours to hire their car. If I do not have money I tell them that I will pay later.”* [Male speaker 29, isiXhosa]

Participants were putting themselves at risk by seeking healthcare: from crime, infections, and treatment delays. Accessing facilities was often burdensome and had indirect costs, participants therefore relied on the help of their community to receive medicines or shared them.

#### Impact of illness on earnings

The impact of their illness on work and income was a key theme that many participants raised. For some the direct and indirect (e.g., transport) costs were so high that they “can’t spend any money on other things” [Female speaker 3, Afrikaans].

The desire to continue paid work was a driver for many to want to get better. This participant enquired:

*“Maybe [the hospital] can help me cure the asthma totally, because now it’s making my life miserable, because I would not be able to work now.”* [Male speaker 27, isiXhosa]

This lady reported that she was unable to claim the advantages of the “sick role” (for example adjusted work duties) and that she was forced to leave work due to her illness:

*“When I worked the woman told me: I think you should go home, you’re coughing. You’re coming for your money and now the woman is telling you to just go home because you’re coughing.”* [Female speaker 9, Afrikaans]

Yet to mitigate the negative effects, some, like this participant, achieved the “sick role” and received support from the doctors and the government, in the form of advice and disability grants:

*“I have to work on “light time” [because of my lungs] and the bosses don’t want that. And that is how it affected my work. I told the doctors also about it, and they said I must quit the job … Because you will only make the problem worse. And that is why I quit it. And that is why they started giving me disability grant.”* [Male speaker 20, English]

However, some, like this participant, were left to educate their employers by themselves, after she was empowered by her own understanding of the illness:

*“I told [my boss, Madame] her that it’s not that I’m an infectiously ill. I told her asthma isn’t an infectious disease. It’s just when I move around too much then I start coughing. I had to tell Madame don’t be surprised if one day I come in and I cough constantly. That’s when I suffer for breath. And don’t be afraid if I take out my pump here in front of Madame and [mimes taking inhaler]. I say that’s when I use it to open the lungs, that’s all.”* [Female speaker 4, Afrikaans]

Family and friends would also help financially, for example this participant’s neighbour:

*“My neighbour that has a car yesterday they said when you have an asthma attack tell us. I told him that I need to go to [large hospital], I do not have money. They gave me money.”* [Male speaker 29, isiXhosa]

The impact of the illness on work was very significant. Some had difficulty achieving the “sick role” and its benefits.

## Discussion

### Main findings

Having a CRD had a substantial impact on the participants. Socio-economic factors, such as working in manual labour or living in sub-standard housing, were perceived as contributors and causes of CRDs. Participants experienced delays in receiving a diagnosis due to multiple, interrelated factors that included inadequate clinical investigations, self-treatment, and limited time with healthcare professionals. This, according to the participants, translated into confusion and possible mismanagement. Larger hospitals with more staff were preferred. They reported that there were putting themselves at risk when seeking healthcare due to street violence and crime as well as risk of hospital acquired infections, as they faced delays in treatment. Attending clinical facilities was often burdensome and had indirect costs, patients reported that they relied on their social networks such as family and neighbours for sharing medicines or transportation to the clinics. Participants paid out of pocket to see professionals, for transport and medicines from private pharmacies. The impact of the illness on work was very significant: Some experienced prejudice or had to change or quit jobs.

Frustrations about the interactions with healthcare professionals by children and their caregivers in the region had been described previously, but participants now revealed that this is also experienced by affected adults themselves [43, 44]. Healthcare workers in Africa have expressed that they encounter challenges to treat those with CRDs, but the effects of these challenges on affected adults themselves had not been described previously [14, 45]. Some participants described how health education empowered them to manage their illness. A systematic review of studies from high-income countries identified interactions with healthcare professionals, and the patients’ understanding of illness and treatment, as modifiable factors to improve care [46]. Our findings suggest that this may also be applicable to LMIC settings.

Given the high unemployment rate within South Africa and among our participants, the impact of disease on paid work was a key theme. Our participants perceived the work environment as contributing to poor lung health. The participants had trouble claiming privileges associated with the “sick role” that can, in some instances lead to ‘paid sick leave’ or other support. Studies from Australia reported that patients pushed their physical limits to attend work even in this high-income setting [47, 48]. In a qualitative study in Sudan and Tanzania, participants reported a spiral of economic loss due to direct costs of medicines and the inability to do physical work due to illness and lack of energy [37]. In this study adults with CRDs in South Africa stated for the first time that they feared that being ill and unable to work would result in long term unemployment and poverty, and that they continued to expose themselves to potentially harmful substances to stay in work.

The experiences of participants living with CRDs were associated with structural barriers to exercising the right to health, reminding us of the work by Galtung and others who developed the ‘structural violence’ concept in the 1960s. This theory was developed, as a way of describing “social structures – economic, political, legal, religious, and cultural – that stop individuals, groups and societies from reaching their full potential” [49]. “Violence” here describes the injury that people experience through uneven access to resources [50]. This is caused by structural factors, such as education, law or the economy, which are often deeply embedded in society [50]. In the context of healthcare, the concept is closely entwined with the social determinants of diseases, including health policies and systems, and the individual’s socio-economic positions and living environments. Whilst structural violence does not solely affect those with CRDs, those who were poor and lived with CRDs in Cape Town were particularly vulnerable to these factors that were beyond their control, especially regarding air pollution and access to emergency treatments. The participants lived in dustier areas due to their socio-economic status and many performed manual work, exposing them to unavoidable pollution. They could not control the violence, or the lack of affordable transport to the facilities, but these precarious and unpredictable risks within their communities could result in delays as they were reliant on others to transfer them to facilities or, in extreme cases, because they had to wait for police escorts and ambulances in life-threatening medical emergencies. Studies from the USA described that violence and poverty had direct effects on childhood asthma severity, and it may be interesting to explore whether this is the case for adults in Cape Town [51, 52].

We also identified that structures which should work in the participants’ favour, such as a national social security system for financial support, or protective policies on air pollution, health or safety were not or inadequately enforced according to the participants [53]. In South Africa the employer is responsible for provision of health and safety, and the use of protective equipment in the workplace [24, 54]. Compensation funds indicate that respiratory conditions form a large part of the mortality from occupational diseases [55]. Yet data on policy enforcement and occupational diseases is scant. Future work could try to establish whether this perception of the participants is indeed true, and how to ensure these protective structures support those with CRDs.

The perceived solutions to the identified barriers were mainly inter-personal. Working with communities and health education may therefore be effective to improve healthcare. It will be important to further investigate and address the habit of medicine sharing that many participants described. Many perceived private healthcare as the solution to their problems. Yet an understanding of the experiences of those with CRDs contributes to addressing health inequities by recognising that perceived “simple” solutions, such as specific medicines or private healthcare, may be inadequate to address the underlying causes and perpetuators of poor health and healthcare. Solutions on multiple levels are needed instead.

### Strengths and limitations

#### Strengths

The perspectives and opinions of 32 adults with CRDs in Cape Town were explored using different local languages. The participants came from a variety of socio-economic and cultural backgrounds. The analysis was triangulated between different researchers with different backgrounds, thereby alleviating the influence of individual researcher’s perspectives and improving rigour. Structural violence as a concept was clearly demonstrated through framing of the experiences of living with CRDs in Cape Town,

#### Limitations

The participants were sampled purposively to allow representation of the DAD-CT study population (Appendix). Healthcare professionals, other stakeholders, and those exclusively using private healthcare were not included in this study.

#### Future research

Most participants were unemployed or pensioners and given that work was such a central theme in the discussions, it would be useful to specifically explore the views of those in employment. To enrich our understanding of the structural influences in CRD healthcare provision healthcare workers could also be interviewed. Ethnographic studies focussing on the pluralistic health-seeking pathways could provide more insights in to how CRDs are interpreted in this setting.

## Conclusions

The experience of living with CRDs and accessing healthcare in Cape Town illustrated clearly how structural violence operates. Whilst seeking healthcare, participants experienced structural barriers, including multiple risks beyond their control at work and in the air (pollution). There were many perceived dangers that participants needed to navigate and overcome to receive healthcare. Actions on multiple fronts are therefore needed to overcome the “bully disease”: For patients and communities this may include making medicines more accessible and affordable, health promotion interventions about the risks of medicine sharing, and community empowerment for peer support. For healthcare facilities, increasing staff numbers, training on CRDs and tests may be helpful. Politically, engaging policy makers to enforce protective air pollution and workplace policies, and to provide adequate financial support for those who find it difficult to work would be valuable.

### Supplementary Information

**Additional file 1: Appendix.**

## Data Availability

Due to potentially identifiable participant information, even where de-anonymised, qualitative data are not publicly available but may be available from the corresponding author on reasonable request.

## Abbreviations

CRD: Chronic respiratory disease
COPD: Chronic obstructive pulmonary disease
LMIC: Low- and middle-income country
WHO: World Health Organization
DAD-CT: Diagnosing Airways Disease in Cape Town study
MS: Marie Stolbrink
MC: Martha Chinouya
CS: Chantel Streicher
KMc: Khanyisa Mcimeli
