# Supplementary material for: “Asthma is a very bully disease” – patient experiences of living with chronic respiratory diseases in Cape Town, South Africa
Source: Int J Equity Health. 2023 Sep 14;22:190. doi: 10.1186/s12939-023-02002-5 (PMC10500759; doi:10.1186/s12939-023-02002-5)
Supplement: Supplementary file 1 — Additional file 1: Appendix. [file 12939_2023_2002_MOESM1_ESM.docx]

Appendix

“*Asthma is a* very *bully disease*” – patient experiences of living with chronic respiratory diseases in Cape Town, South Africa. Stolbrink et al. 2023.

Contents

[2 Standards for Reporting Qualitative Research (SPQR) Checklist 1](#_Toc136012089)

[3 Topic Guide for participant interviews 3](#_Toc136012090)

[4 Overview of participants in DAD-CT study and interviews 5](#_Toc136012099)

# Standards for Reporting Qualitative Research (SPQR) Checklist

According to: Standards for Reporting Qualitative Research: A Synthesis of Recommendations.

O’Brien, Bridget C.; Harris, Ilene B.; Beckman, Thomas J.; Reed, Darcy A.; Cook, David A.

Academic Medicine89(9):1245-1251, September 2014. doi: 10.1097/ACM.0000000000000388


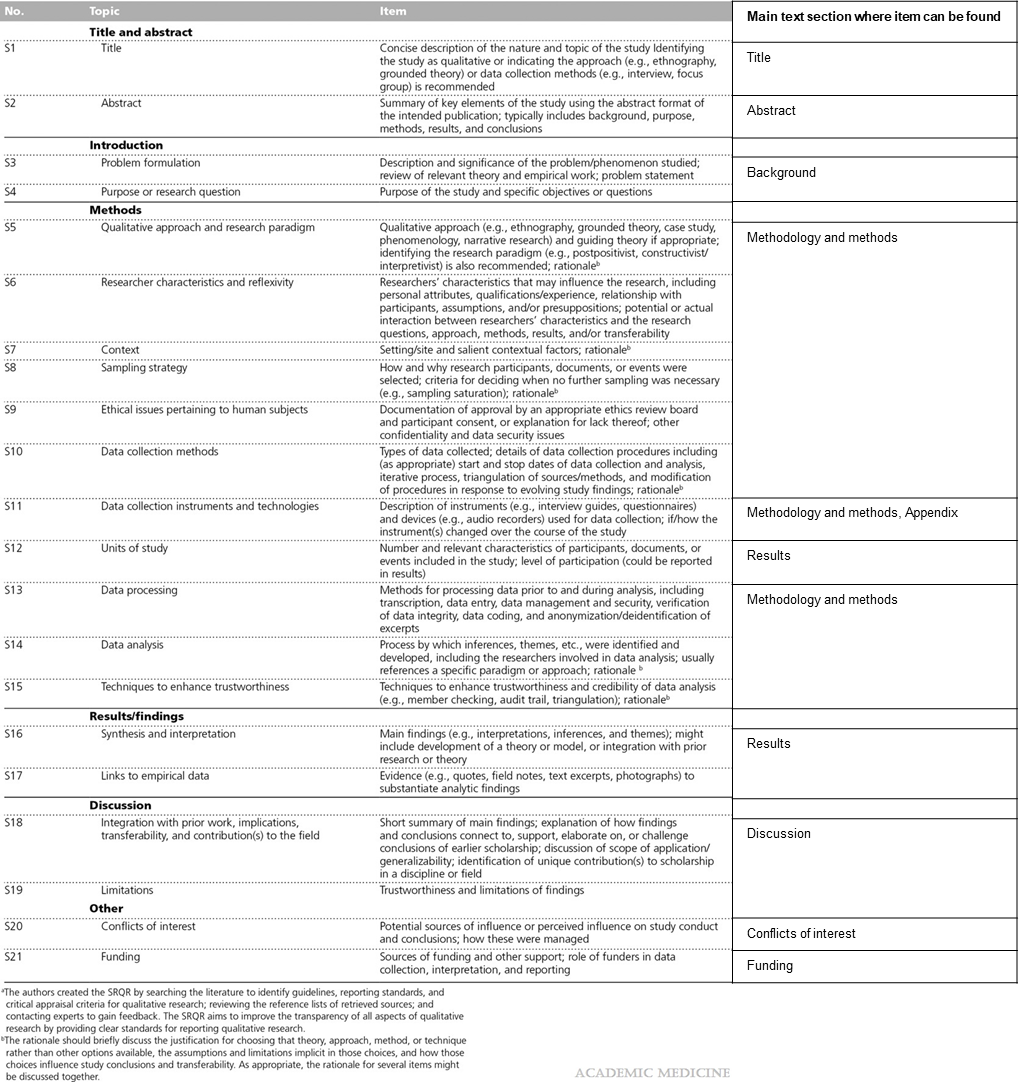


# Topic Guide for participant interviews

Preamble (to be read by the facilitator):

Today is the [insert date] and it is [insert time XX:XX]. This is a discussion with a patient participant that forms part of the “Airways disease in a high TB and high HIV prevalence setting – making the correct diagnosis and investigating accessibility to care” study. Briefing for the interview.

[Introduction of the interviewer]

Thank you for agreeing to take part by signing the consent form. Are you still happy to proceed?

Just to remind you, this interview is for a research study about chronic lung disease and understanding the experience of both those with chronic lung disease and others involved in their care. We greatly value your perspective. There are no right or wrong answers to the questions we will ask. The interview will take up to one hour.

I would like to digitally audio-record our conversation using this device. This will allow us to type our conversation, but any names or places that you mention will be taken out so that you will be anonymous. No-one can identify you. This way, if someone were to read this interview, they would not be able to know who you are because your name will be replaced with a code which is a number. You can choose not to answer a question at any point during the interview.

Do you have any questions before we go ahead with the interview?

The following are examples of questions, themes and probes that should be used during the interview.

## Participant background

I would like to start by asking some questions about your background:

- Note the sex (woman or man); age, where they live
- How did you get to be part of the study?
- Place of birth; year.
- Marital status; children; living arrangements (household members, type of housing); check cooking in the house; electricity.
- Employment: probe.
- What is your neighbourhood like? Is it safe?

## Feeling unwell

- How did you get to know you have chest problems?
- Year
- What happened when you started feeling this chest problem the last time? What symptoms did you have? How did it start?
- Where did you go when it started? Why there?
  - Probe: Feelings; symptoms, distance, fees, skills?
- What did you think was the cause of this? What made it worse? What did you do to feel better?
- Does anyone else in your family have the same chest problems?

## Going to the hospital with the emergency chest problem

- How long after the development of the chest problem did you come to the hospital?
- Did you go anywhere else first (clinic, pharmacy …)? Where? Why?
- What made you think of coming to the hospital?
  - Probe: distance, transport; money; seriousness of symptoms? Suggestions from others?
- When you came to the hospital what did the doctors / nurses say you have? Who told you? Examples: asthma / COPD / post-TB lung disease. How did you feel?
- What did they say you have to do about it?

## Making sense of diagnosis and treatment

We would like to ask you more about your experiences of becoming sick with chronic lung disease / asthma / COPD / post-TB lung disease [delete as appropriate, use the diagnosis given by the participant]. So, the doctor says you have … - What does that mean to you? What causes it?

- Tell me more about first being told (diagnosed) with … Who told you? How did you feel when you were told?
- What did the professionals say was the cause of your chest problems? Do you agree with them?
- Who [professionals] normally looks after your chest with you? Nurses? Pharmacists? Doctors? What do they do?
- How do you access the medicines? Any issues that you experience in accessing the medicines or seeing the doctor/nurses?
- What do you understand about the treatment of the condition?
- What treatments / drugs do you use for your lung disease? Where on the body? Where do you get the medicines? How accessible?
- Please tell us about how you use your treatment? What time? What did the professional tell you to do with the medicine? Do you fully follow what the doctors/nurses told you to do? Why? What do you think of the treatment?
- How do you think each of the medications work? (Where in the body?)
- Do you use other methods of treating your lung disease? Like herbs? How do you get them? How did you find out about them?
- How do you feel when using the medication? What do you think other people think of you when you are using the medication?
- Do you get any side effects or unwanted effects from your medicines? How does that impact you taking them?

## Impact on daily life

- How does having the lung disease make you feel day to day? How does it affect your work? How does it affect your family?
- Do the medicines affect your daily life? How? Why? What do you do?
- On a day to day basis, how do you look after your lungs? What do you do? Why?
- What do you do when the illness is flaring up or you have trouble breathing? Probe: work; looking after family; who helps, why.
- Who know about your illness? Who have you told about the illness (why?)? Do you mind others know about your illness? Why?

## How to improve services

- Tell me how we can improve services
- What things influence how well you can look after your lungs / breathing?
- Probe sensitively: e.g. stigma, psychological impact, financial impact
- What makes it easier for you to receive care for your lung disease?
- What prevents you from receiving care for your lung disease?
- If you could change one thing in the care of your lung health what would it be? What additional treatment would you like to receive? What other support would you like to receive?

## Covid-19

- What impact did the pandemic have on your lung health? Did it make anything better? Did it make anything worse? Did it influence your or other people’s ideas / stigma about lung health?
- What can we do to prepare for future waves or pandemics?

## Closing

Thank you so very much for taking the time to talk to us today. Is there anything else we need to know? Do you have any questions?

# Overview of participants in DAD-CT study and interviews

|  | **DAD-CT study** | **Interviews** |
| --- | --- | --- |
| **Attribute** |  | **Number (percentage)** |
| **Total number** | 63 | 32 |
| Gender |  |  |
| - Women - Men | 33 (52 %)  30 (48 %) | 17 (53 %)  15 (47 %) |
| Age |  |  |
| - > 50 years - ≤ 50 years | 49 (78 %)  14 (22 %) | 25 (78 %)  7 (22 %) |
| Preferred language |  |  |
| - Afrikaans - isiXhosa - English | 33 (52 %)  17 (27 %)  13 (21 %) | 16 (50 %)  8 (25 %)  8 (25 %) |
| Employment |  |  |
| - Employed - Unemployed - Disability grant - Pensioner | 7 (11 %)  26 (41 %)*  Not recorded  30 (48 %)* | 3 (9 %)  13 (41 %)  5 (16 %)  11 (34 %) |
| Household |  |  |
| - Lives alone - Lives with others | Not recorded  Not recorded | 3 (9 %)  29 (91 %) |
| Relationship status |  |  |
| - In relationship / married - Single / widowed | Not recorded  Not recorded | 14 (44 %)  18 (56 %) |

*In the DAD-CT study a disability grant could have been included in either unemployment or pensioner categories.
